# Adapting the Nominal Group Technique to a virtual version: an experience report

**DOI:** 10.1590/1980-220X-REEUSP-2023-0298en

**Published:** 2024-03-11

**Authors:** Viviane Cristina de Lima Gusmão, Tatiane Garcia do Carmo Flausino, Daniela Sanches Couto, Ligia Maria Abraão, Adriana Maria da Silva Felix, Caroline Lopes Ciofi-Silva, Molly Courtenay, Valerie Ness, Enrique Castro-Sanchez, Rosely Moralez de Figueiredo, Maria Clara Padoveze

**Affiliations:** 1 Universidade de São Paulo, Escola de Enfermagem, Departamento de Enfermagem em Saúde Coletiva, São Paulo, SP, Brazil.; 2 Universidade Federal de São Carlos, Departamento de Enfermagem, São Carlos, SP, Brazil.; 3 Universidade Estadual de Campinas, Faculdade de Enfermagem, Campinas, SP, Brazil.; 4 Cardiff University, Cardiff, UK.; 5 Glasgow Caledonian University, Glasgow, Scotland, UK.; 6 Brunel University London, London, UK.; 7 University of Balearic Islands, Palma, Spain.

**Keywords:** Nursing, Nursing Research, Digital technology, Methods, COVID-19, Enfermería, Investigación en Enfermería, Tecnología Digital, Métodos; COVID-19, Enfermagem, Pesquisa em Enfermagem, Tecnologia Digital, Métodos, COVID-19

## Abstract

**Objective:**

To report on the adaptations made to the original Nominal Group Technique (NGT), allowing it to be applied to the virtual format, preserving all its key elements.

**Method:**

An experience report on the adaptations and adjustments made to the original NGT to the virtual format using Information and Communication Technologies (ICT), using digital tools that are available free of charge or are low cost and easy to use.

**Results:**

The NGT was carried out entirely virtually and underwent adaptations in each of its four stages through the incorporation of specific digital resources. It was possible to present the most voted ideas and obtain final approval from the participants. The participants had no difficulty in using the virtual resources provided and, based on the reaction evaluation, they were satisfied with the tools provided.

**Conclusion:**

The adapted NGT proved to be an effective method when used in a virtual setting, capable of producing a significant number of ideas and developing consensus. The adapted tool can be used by other researchers in countries with similar resources or dimensions to Brazil.

## RESUMO

**Objetivo:**

Relatar as adaptações realizadas na Técnica de Grupo Nominal (TGN) original, permitindo sua aplicação ao formato virtual, preservando todos os seus elementos-chave.

**Método:**

Relato de experiência sobre as adaptações e adequações realizadas na TGN original ao formato virtual aplicando as Tecnologias da Informação e Comunicação (TIC), por meio de ferramentas digitais disponibilizadas gratuitamente ou de baixo custo e de fácil manejo.

**Resultados:**

A TGN foi realizada integralmente de forma virtual e sofreu adaptações em cada uma das suas quatro etapas através da incorporação de recursos digitais específicos. Foi possível apresentar as ideias mais votadas e obter a aprovação final dos participantes. Os participantes não apresentaram dificuldade para utilizar os recursos virtuais disponibilizados, e, partir da avaliação de reação, mostram-se satisfeitos com as ferramentas disponibilizadas.

**Conclusão:**

A TGN adaptada mostrou-se um método efetivo quando utilizada em cenário virtual, sendo capaz de produzir um significativo número de ideias e desenvolver consenso. A ferramenta adaptada pode ser usada por outros pesquisadores em países com recursos ou dimensões semelhantes ao Brasil.

## RESUMEN

**Objetivo:**

Informar sobre las adaptaciones realizadas a la Técnica de Grupo Nominal (TGN) original, permitiendo su aplicación al formato virtual, preservando todos sus elementos clave.

**Método:**

Se trata de un informe de experiencia sobre las adaptaciones y ajustes realizados a la TGN original para el formato virtual mediante el uso de las Tecnologías de la Información y la Comunicación (TIC), utilizando herramientas digitales disponibles de forma gratuita o de bajo coste y fácil uso.

**Resultados:**

El TGN se realizó íntegramente de manera virtual y sufrió adaptaciones en cada una de sus cuatro etapas mediante la incorporación de recursos digitales específicos. Fue posible presentar las ideas más votadas y obtener la aprobación final de los participantes. Los participantes no tuvieron dificultades para utilizar los recursos virtuales proporcionados y, según los comentarios recibidos, se mostraron satisfechos con las herramientas facilitadas.

**Conclusión:**

El TGN adaptado demostró ser un método eficaz cuando se utiliza en un entorno virtual, capaz de producir un número significativo de ideas y desarrollar el consenso. La herramienta adaptada puede ser utilizada por otros investigadores en países con recursos o dimensiones similares a las de Brasil.

## INTRODUCTION

The Nominal Group Technique (NGT) is a highly structured methodological approach for research with groups, used to explore themes and develop consensus^(1)^. This method allows a group to identify, classify and assess needs in a problematic area without interference from researchers^(2,3)^.

This technique brings together a group of people with common goals to discuss a problem and produce ideas. Prioritization methods can vary, but in the end, everyone arrives at a set of ideas that represent the group’s consensus. NGT has been improved and used to define priorities for new research^(2,3)^.

The greatest advantages of NGT are the possibility of social interaction and democratic discussion of the guests, the limitation of the researcher’s influence and time efficiency, due to the opportunity to acquire a high yield of data in a relatively short period of time. Other advantages are related to its low cost and ease of adaptation to various contexts^(1,4)^. Compared to the Delphi Technique, NGT has a lower drop-out rate throughout the process^(5,6)^.

In addition, aspects relating to the high costs involved in the displacement of participants in a country of continental dimensions such as Brazil, and easier access to new information and communication technologies (ICT) made it possible to adapt the NGT to a virtual format^(7)^.

The COVID-19 pandemic has affected researchers all over the world, leading to the need for adjustments and adaptations in numerous research designs and in different circumstances^(8)^. For example, we were suddenly unable to hold the face-to-face meetings required for an international workshop planned using the NGT. This workshop aimed to reach a consensus on the main research gaps in the topic studied, the role of nurses in antimicrobial stewardship programs, and to list priorities for future research.

This study reports on the necessary adaptations made to the traditional NGT, allowing it to be applied to the virtual format, preserving all its key elements, as well as overcoming challenges related to this process. We believe that sharing this experience will contribute to the innovation of methodologies that can be adopted to identify gaps in other fields of nursing research.

## METHOD

### Design on Study

This is an experience report describing the necessary adaptations made to the NGT for the virtual scenario.

### Local

The adapted NGT was used during the workshop “The Role of Nursing in Antimicrobial Stewardship Programs”, which was designed to identify research gaps on the subject in the Brazilian scenario and proposals for new collective research projects.

The workshop was designed to take place over 3 days, with intervals of approximately 2 weeks between them, and was intended to be attended by up to 30 nurses working in different healthcare contexts in the five regions of Brazil, as well as the project’s executive team. The participants were predominantly female, aged between 30 and 63. They had between 8 and 39 years’ professional experience, with the most prevalent work in hospital environments (n = 11 nurses, 42.3%), followed by professionals working in academic environments developing research (n = 6, 23.1%). The majority of participants (n = 20 nurses, 73.1%) had no experience of the NGT method. Data collection took place during the months of May and June 2022.

### Adaptation Procedures

The adaptation was carried out in such a way as to preserve all the key elements of NGT and avoid mischaracterizing it. The four stages of traditional NGT were identified from the literature (Chart 1) and the adaptation process was planned to ensure that these stages were fully developed. The need to restrict costs also determined the choice of tools that were freely available and could be easily handled without the support of information technology personnel. Resources traditionally used in NGT, such as the use of multicolored self-adhesive labels (post-its), blackboards, flip charts, meeting rooms with tables and chairs and sketch papers, were also the focus of the search for virtual alternatives, with the aim of simulating face-to-face situations as much as possible.

**Chart 1:** Description of the stages of traditional NGT – São Paulo, SP, Brazil, 2022^(1)^.

Stages	Description
*Generation of ideas*	The facilitator guides the participants to write down their ideas in brief phases or statements according to the guiding question using a blank sheet of paper, cards or post-it notes.
*Presentation of ideas*	Each participant presents, without opening discussion, one of the ideas on their list, in rounds of presentations. The ideas are recorded on flip charts or post-it notes by the supporter for everyone to see.
*Clarification of ideas*	The participants express the relative importance of each idea. If there are duplicates, the ideas can be combined with the agreement of the group.
*Voting*	Participants vote privately to prioritize the ideas, using criteria created by the facilitator. The facilitator will add the scores assigned. The results are then discussed in the group and recorded by the supporter, with the ranking order of the issues and concerns identified.

Source: Michel et al.^(1)^

**Figure 1:**
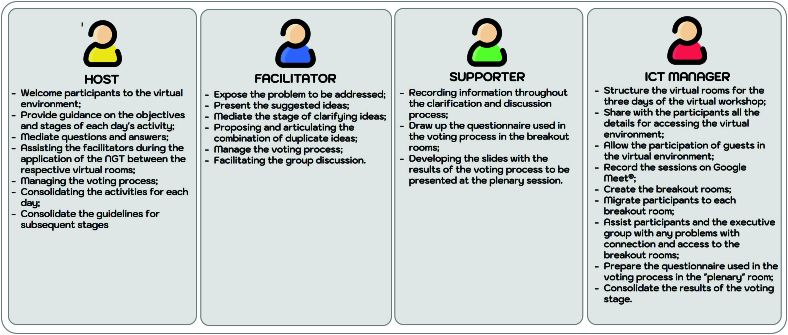
Roles of the executive team during the Nominal Group Techniques, São Paulo, SP, 2022.

Source: Authors, 2022.

Note: ICT: Information and Communication Technology.

The workshop was planned by an international executive team made up of nurse researchers from the UK and Brazil. The members of the executive team had previous experience of using the traditional face-to-face model of NGT and worked to identify virtual technological alternatives for applying the stages of virtual NGT. The roles of each member of the executive team were defined as shown in Figure 1. Due to financial restrictions for simultaneous translation, only the Brazilian members of the executive team worked on the application of NGT.

### Evaluation of Reaction

A reaction evaluation form was constructed, which covered aspects about the topic, the virtual setting, the workshop, the performance of the facilitators and supporters, and a self-assessment. Participants were emailed a link to access the electronic form containing objective questions and a five-point Likert scale (0 – very unsatisfactory | 1 – unsatisfactory | 2 – satisfactory | 3 – totally satisfactory | 4 – No opinion).

### Ethical Aspects

This research was cleared by the Research Ethics Committee of the USP School of Nursing under Opinion No. 5.381.334 of 2022, and was carried out following the ethical precepts in force according to Resolution 466/12. As the data was collected in a virtual environment, all the requirements for research procedures with any stage in a virtual environment were followed, according to Circular Letter 01/2021/CONEP/SECNS/MS and Law No. 13,709, of August 14, 2018, which provides for the protection of personal data. All data relating to this research was stored on a personal computer with a login and password that was not shared. No cloud records were kept. The informed consent form was sent via email and signed by all participants before the workshop took place.

## RESULTS

**Figure 2:**
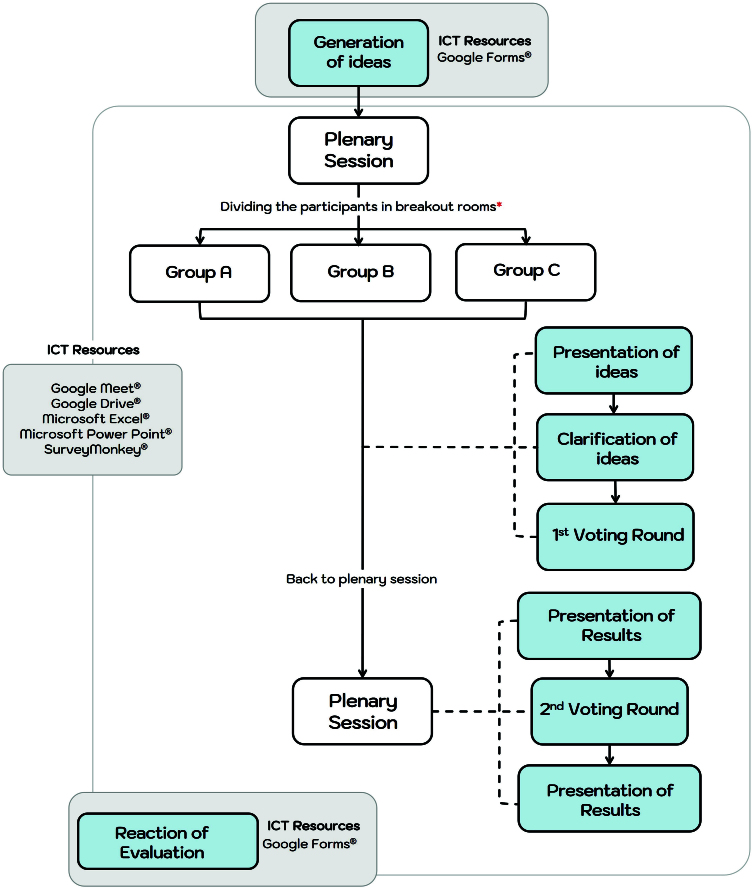
Schematic representation of the virtual displacement of the participants and the digital resources used.

Source: Authors, 2022.

Note: *Google Meet^®^ can be divided into several breakout rooms. The number of rooms was adapted to the needs of the research and the number of participants in the NGT.

The adaptation of each stage of the NGT required the incorporation of specific ICT resources, as shown in Figure 2. These resources were acquired free of charge and managed by the executive team, thus implying no additional operating costs. All project planning and execution activities took place remotely, with no travel or accommodation costs. The number of participants was not a limiting factor for the virtual adaptation, since most of the platforms currently available are compatible with the number of participants envisaged for this type of activity. The choice of tools/resources was made taking into account the following elements:

– Ease of access and use of Google®-based platforms, since they are widely used in the country;
– Ease of use by the executive team without specialized technical support;
– High audio and video quality;
– Sending invitations by e-mail and automatic inclusion in participants’ calendars and schedules;
– Sharing of files and documents relevant to the activity;
– Sharing of the main room in breakout rooms;
– Recording and documenting the processes and results of activities: recording of sessions to enable the process to be traced (e.g. number of participants who voted); enabling graphs and analysis of results to be drawn up quickly;
– Holding meetings without limits on participants and/or time;
– Access to voting links and applications via individual mobile phone;
– Possibility of anonymity for the idea generation and voting processes;
– Possibility of shared editing of documents during the idea clarification stage.

The roles of each member of the executive team were defined as shown in Figure 1. The development of the adapted NGT stages is described in detail below (Figure 2). At the start of each activity, the participants were instructed on the procedures to be adopted in the virtual environment, with special attention to the mode of transition between the virtual rooms, managed by one of the members of the executive team. All the results, bibliographical references and presentations related to the NGT adapted in this study were shared with the participants via a virtual file storage and synchronization system, ensuring transparency at all stages and encouraging participants to access the content made available.

*Stage 1: Generation of Ideas.* The introduction and contextualization of the topic, as well as the presentation of the research proposal, took place on the first day of the workshop, held virtually via the Google Meet® platform. At the end of the event, participants were instructed on the next stage, idea generation, in which they received, via e-mail, a link to an electronic survey form prepared using the Google Forms®, containing the guiding question for the NGT. Participants could identify themselves using a nickname in order to keep their ideas confidential. As such, the silent idea generation stage, normally done in person, was replaced by the remote activity, with the advantage of allowing additional time for reflection by the participants. Participants were given approximately 10 days to individually register up to three ideas on the electronic form sent to them. The ideas should address the main research gaps on the guiding question presented, from the point of view of each participant according to their work context. All the ideas recorded were organized in a spreadsheet using Microsoft Excel® software. The prepared file was shared with the executive team via Google Drive®, with a view to pre-analyzing the content.

*Stage 2:* Presentation of ideas. This took place on the second day of the workshop. Participants received a link to the Google Meet® platform and accessed the main virtual room, called the “plenary”. The host explained the importance of writing research questions and the use of the FINER criteria (feasible, interesting, new, ethical and relevant)^(9)^, so that participants could convert the ideas registered on the electronic form into relevant research questions. The participants were randomly “allocated” into three groups (A, B and C) and each group discussed only the ideas previously generated by its participants. The option of dividing into groups took into account the excessive time and fatigue that could be generated by discussing all the ideas in plenary with all the participants. The choice of groups also favored greater opportunity for individual expression and interaction between the participants. Each group had a facilitator and a supporter. The facilitator shared on screen and presented the original ideas generated by the group, organized in a Google Drive® spreadsheet so that everyone could see them.

*Step 3: Clarification of ideas.* The facilitator made it clear that all the original ideas would be converted into research questions during the clarification stage. Through the coordination of the facilitators, the groups made changes to the wording, such as grouping similar ideas together, deleting duplicate ideas and finally converting the ideas into research questions. The supporter recorded the adjustments by consolidating the spreadsheet in Google Drive®.

*Stage 4: Voting.* The voting stage to prioritize the ideas was carried out in two rounds using the SurveyMonkey® online platform, a questionnaire model with multiple alternatives, in which each alternative consisted of a research question that was a candidate for a vote. Groups A, B and C received the voting links for their respective survey questions via Google Meet® chat, and were instructed to use their cell phones to access the survey link and vote individually and anonymously. In the first round of voting, the participants in each group were instructed by the facilitators to select up to three research questions that they considered important. At the end of the first round of voting, the result with the three research questions most voted for by each group was presented by the respective facilitator for approval by the participants. After this stage, all the participants and the executive team were redirected to the “plenary” room, where each facilitator presented their group’s results. Nine research questions were then presented in a second clarification phase, followed by a second round of voting, in which the three research questions they considered most important were democratically elected. Due to the limited number of participants in the free version of the tool available on the SurveyMonkey® platform, it was necessary to carry out the second round of voting fragmented into blocks according to the respective groups A, B and C, for which specific links were generated, but containing the same ideas for voting. The results of the three voting blocks were aggregated for the final tally of votes. The three research questions with the most votes in the final round of voting were presented in plenary for final approval by the participants.

Throughout the process there were no difficulties for participants in interacting using the virtual tools to request to speak; participants had no difficulty moving between the plenary and breakout rooms; the time allocated for each activity was fulfilled according to plan.

The reaction evaluation form was answered by 100% of the participants (26). In the general evaluation of the digital technologies used, 88.5% (23) of the participants considered the digital resources to be completely satisfactory. Access to the Google Meet® platform, access to the sharing drives and filling in the form via Google Forms® were also considered totally satisfactory by 88.5% (23) of the participants. As for the voting system via SurveyMonkey®, although it was considered satisfactory by the majority of participants, it had a lower approval rate, since 84.6% (22) of the participants considered the resource used to be totally satisfactory.

## DISCUSSION

NGT, like other research designs, is highly dependent on the formation of groups and interviews, which became unfeasible due to the physical isolation adopted in the context of the pandemic and had to undergo adaptations^(10,11)^.

The use of ICTs was the solution found to continue the interrupted actions, including teaching and research. This high demand, in turn, contributed to increasing the availability of the necessary tools for free or at a very low cost, and stimulated the accelerated growth of the population’s digital education. Some authors believe that these actions have contributed to increasing the population’s digital inclusion and that it will persist after the pandemic^(11,12)^. Currently, studies show that there is a movement from online teaching practices to face-to-face education using the benefits of ICT for learning^(13,14)^.

The number of studies describing the advantages of using ICT in the data collection process in qualitative research is still incipient^(15)^. This is particularly important when we talk about studies that apply NGT, since when making adaptations to the method there is a risk of deviations from the traditional method. Even so, there is an incentive for future studies to consider these adaptations in order to make qualitative studies more accessible^(16)^. The literature already presents studies that have applied the NGT adapted due to the context of the pandemic^(1,17,18)^. However, these studies do not describe in detail what adaptations were made to the method^(19,20)^.

Even before the pandemic, the proposal for an online version of the NGT has been discussed, and was effectively applied in a study carried out in April 2018 with patients living with a chronic condition. The study highlights that four sessions of the NGT were carried out, three of which were online, and only one of which was face-to-face^(20)^, unlike the present study, which was designed and carried out entirely in virtual format, without the need for a face-to-face meeting. When comparing the adaptations in the studies, it would be possible to increase interaction between the participants with a day of face-to-face activities, but costs would be an obstacle.

In another study, to assess the feasibility and acceptability of the NGT fully adapted for a virtual version, the researchers used the Zoom® platform to hold pre-scheduled meetings. Microsoft Excel® and Microsoft Word® were also used to record the ideas. Voting and ranking of the ideas was done using the Mentimeter® tool, the free version of which is limited to 10 participants and requires paid specific plans for a larger number of participants^(21)^.

The authors pointed out various challenges encountered in using the proposed ICTs. Many participants had difficulties with chat on the Zoom® platform, as well as editing Microsoft Word® and Microsoft Excel® files, when using mobile access. In the proposed adaptation, participants were responsible for recording their ideas in the platform’s chat, which were then transferred to Word and Excel files. Despite the challenges encountered, the researchers positively assessed the feasibility and acceptability of the NGT adapted for the virtual version^(21)^.

On the other hand, in our study, the participants did not point out similar difficulties regarding the use of the available ICT, since, in the general evaluation of the ICT used, 84.6%^(22)^ of the participants considered the digital resources to be completely satisfactory. The level of digital literacy of the participants was not investigated in order to assess the extent to which the use of ICT may have positively or negatively affected the interaction. However, the reaction assessment indirectly showed that, despite the adaptation of this method, the participants’ ability to understand, interpret and interact in the virtual environment was not impaired. It is important to note that, during the clarification of ideas stage, the participants and the facilitator were free to debate, clarify and give their opinion, without having to worry about recording the changes in a file, since this activity was the responsibility of a supporter in each of the thematic rooms. This approach may have helped this stage to run smoothly without delays in the schedule and without overloading the participants.

The pandemic-driven approach to ICTs may have contributed to our workshop participants having no difficulties in using them. Many of the ICTs used were already being widely used in other situations, which meant that the participants were already familiar with the digital resources used to adapt the NGT, and this was no longer a major barrier.

By describing the adaptations made to the NGT carried out in this research, we hope to help ensure that the adapted technique can be used by other researchers in similar scenarios and contexts. Each technological resource used has a free or low-cost version, so that costs are not a limiting factor in the application of the adapted NGT. Thus, the adaptation process requires planning and attention to the ICT tools chosen, as there may be a need to purchase a commercial version that meets the needs, with more participants and the need to record the sessions. Another point concerns the privacy of the participants, and it is important that the research meets the ethical standards laid down for data collection in a virtual environment.

As an example, a successful experience with the use of technology during the pandemic was the creation of an interactive global knowledge network, in a virtual model, with a series of webinars on Infection Prevention and Control against COVID-19, carried out through digital technologies and which brought together a group of experts in the field, with an average audience of 634 participants per meeting from 100 different countries, reaching a larger audience than many face-to-face events^(22)^.

Similarly, by using the NGT adapted for the virtual version, this research enabled researchers from all regions of the country to contribute to the issue in question. Considering that Brazil is a country of continental dimensions, this approach allows researchers from different regions to interact without incurring the associated costs inherent in face-to-face events. These costs are often a limiting factor in scenarios where resources and investment in research are scarce.

We should also highlight the current national scenario for nurses, where the excessive workload is another limiting factor for their participation in group research. Holding a virtual event not only saves financial resources but also optimizes the time spent on the activity. Therefore, a flexible model can have a positive impact on the adherence and participation of nursing professionals in consensus research on a wide range of topics of interest.

There were some limitations to the adaptation process. Depending on the ICT tools or platforms chosen, it may be necessary the purchase of a version that meets the needs, depending on the number of participants and the need to record the sessions. Not all participants were able to debate and/or vote on all the ideas, as they were allocated to themed rooms. However, as an operational matter for NGT, it was decided to form groups of no more than 10 participants. As an experience report, it is difficult to generalize data referring to a specific experience, but we believe it is possible to replicate the method’s adaptations for populations and contexts with limited resources.

Therefore, we hope that our detailed description of the adaptation of NGT to a virtual environment will help to design international and national partnerships of researchers, create collaborative networks, optimize financial resources and time. This could benefit not only the field of nursing, but also different areas of research in countries with similar resources to Brazil or in other situations where geographical travel is not feasible or affordable.

## CONCLUSION

The adaptations to traditional NGT described in our report have made it possible to overcome the challenges encountered during the COVID-19 pandemic, resulting in a research design adapted to the virtual modality that can be used in future situations.
